# Coherent destruction of tunneling in chaotic microcavities via three-state anti-crossings

**DOI:** 10.1038/srep04858

**Published:** 2014-04-30

**Authors:** Qinghai Song, Zhiyuan Gu, Shuai Liu, Shumin Xiao

**Affiliations:** 1 Department of Electronic and Information Engineering, Shenzhen Graduate School, Harbin Institute of Technology, Shenzhen, 518055, China; 2 National Key Laboratory of Tunable Laser Technology, Institute of Opto-Electronics, Harbin Institute for Technology, Harbin, 150080, China; 3 Department of Material Science and Engineering, Shenzhen Graduate School, Harbin Institute of Technology, Shenzhen, China, 518055

## Abstract

Coherent destruction of tunneling (CDT) has been one seminal result of quantum dynamics control. Traditionally, CDT is understood as destructive interference between two intermediate transition paths near the level crossing. CDT near the level anti-crossings, especially the “locking”, has not been thoroughly explored so far. Taking chaotic microcavity as an example, here we study the inhibition of the tunneling via the strong couplings of three resonances. While the tunneling rate is only slightly affected by each strong coupling between two modes, the destructive interference between two strong couplings can dramatically improve the inhibition of the tunneling. A “locking” point, where dynamical tunneling is completely suppressed, has even been observed. We believe our finding will shed light on researches on micro- & nano-photonics.

The coherent control of quantum dynamics has attracted considerable research attentions due to their important applications in nano-scale solid state physics^1^, trapped atoms in Bose-Einstein condensation^2^, localized spins in molecular magnets^3^, and Copper pairs in Josephon qubits^4^. Within all these studies, CDT has been one of the remarkable results. In 1991, Grossmann et al. theoretically predicted the suppression and even complete suppression of the tunneling for the first time^5^. The latter case is also known as “locking”. Soon after, CDT has been experimentally observed in several experiments involving cold atoms in double-well potentials^6^ and Mott-superfluid transition in ultracold systems^7^. To date, CDT has been widely applied in a variety of research areas in solid state physics.

The conventional understanding of the CDT lies in the destructive interference between the transition paths for repeated Landau-Zener level crossings^5^,^8^,^9^,^10^. Very recently, with the developing of cavity quantum electrodynamics (cQED) in superconductor circuits^11^,^12^, CDT has also been predicted for the case with ultrastrong coupling and extreme driving^13^. However, the CDT, especially the “locking” that is formed by strong coupling with moderate coupling strength, has not been thoroughly studied. Such kind of study is highly in demand and closely relates to many research areas such as modulating the emissions of semiconductor microcavity and the coupling in plasmonics. The former one will be discussed below. One example of the latter one is the local field enhancement in photonic dimer, where strong coupling with moderate coupling strength usually happens^14^. In general, the field enhancement of photonic dimer increases dramatically with the decreasing of separation distance *w*. Recent developments show that such enhancement has a quantum limit due to tunneling effect when the separation distance is below a critical value *w* < *w_c_*^14^. Therefore, suppressing or inhibiting such quantum tunneling can be a necessary way to further improve the local field enhancement and will boost the whole research direction. Below, we will take chaotic cavities as examples to discuss the possibility to inhibit the tunneling around anti-crossings with moderate coupling strength.

## Results

### The physical model for mode coupling

**Figure 1:**
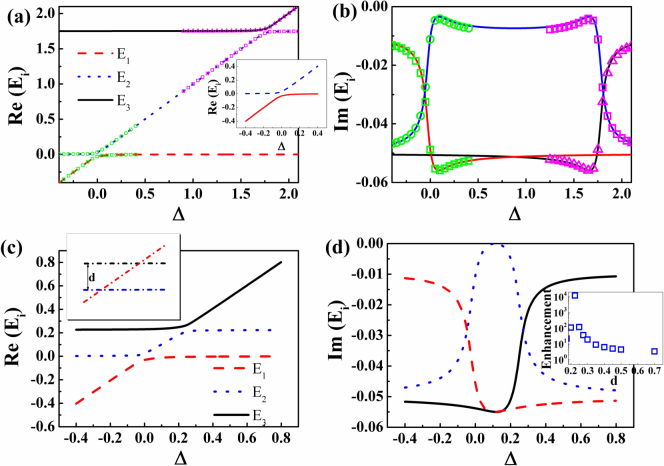
The real (a) and imaginary (b) parts of the eigenvalues of Eq. (3) as a function of Δ. Here *V*_1_*W*_1_ = 0.001 + 0.001118*i*, *V*_2_*W*_2_ = 0.001 − 0.001118*i*, *E*_1_ = Δ − 0.01*i*, *E*_2_ = −0.05*i*, *E*_3_ = *E*_2_ + *d* = *d* − 0.05*i*, and *d* = 1.75. The symbols around Δ = 0 and Δ = 1.75 are the eigenvalues of two-state model in Eq. (1) with *VW* = 0.001 + 0.001118*i* and 0.001 − 0.001118*i*, respectively. For clear view, the anti-crossing of two state model around Δ = 0 is plotted as inset in Fig. 1(a). (c) and (d) are the same as (a) and (b) except for *d* = 0.225. Inset in (c)is the schematic picture of three states without coupling. And the inset in (d) summarizes the dependence of enhancement in lifetime on the separation distance *d*. Here we set *d* ≥ 0.2, which is 2 times larger than |*Im*(*E*_2_)| + |*Im*(*E*_3_)| and makes states -2 and -3 to be well separated in frequency.

Before moving to the real system, we would like to briefly introduce a simple model of avoided resonance crossings. Quantum mechanically, when two states approach each other, their interaction can be descried by a 2 × 2 matrix^15^,^16^

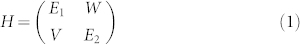
where *E_i_* is the energy of state whose imaginary part determines the lifetime of quasibound state (*τ* = 1/|*Im*(*E_i_*)|), and VW is the coupling constant. The eigenvalues of Eq. (1) can be written as 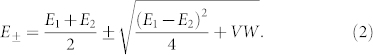
Depending on the coupling strength *VW*, two scenarios can be identified. One is weak coupling with level crossing in *Re*(*E*). The other one is strong coupling with level anticrossing in *Re*(*E*). From Eq. (2), it is easy to get that the lifetime of quasibound state can be influenced by the complex valued *VW* (external coupling) in both scenarios^16^,^17^,^18^. Below we will focus on the strong coupling with moderate coupling strength. One example with *VW* = 0.001 + 0.001118*i* is shown as open symbols in Fig. 1(a) and (b). Around the anti-crossings in *Re*(*E*), the maximum of *Im*(*E*) in Fig. 1(b) is only slightly larger than *Im*(*E*_1_), giving a small enhancement factor in lifetime only about 2. Suppressing *Im*(*E_i_*) to 0 is do possible but requires larger coupling constant that is hard to be fulfilled in practical applications. For a given moderate coupling strength, finding a new method that improves the suppression of *Im*(*E*) turns to be very essential.

For most of complex systems, there are usually more than two states in an interesting energy range. Then one state has the possibility to interact with multiple states at different energies, and these mode couplings have the possibility to interact with each other via interference. Such interference gives a new method to tailor *Im*(*E_i_*). For simplicity, we only consider a three-state model as schematically illustrated in the inset of Fig. 1(c), where state-1 couples to state-2 and state-3 individually. The coupling between states -2 and -3 is neglected by assuming two states are well separated in frequency. Then the interactions within three states in open systems can be understood in term of a 3 × 3 non-Hermitian Hamiltonian 
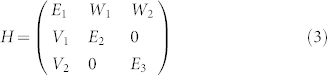
where *E_i_* is the complex energies of states 1–3, *V*_1_*W*_1_ and *V*_2_*W*_2_ are the coupling constants between states 1,2 and states 1,3, respectively. Then the eigenvalues of Eq. (3) can be simply calculated.

When the separation distance *d* is much larger than 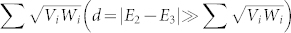
16, the three-state model can be simplified into two two-state models with 
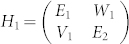
 and 
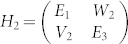
. Taking *V*_1_*W*_1_ = 0.001 + 0.001118*i* and *V*_2_*W*_2_ = 0.001 − 0.001118*i* as an example, the behaviors of the eigenvalues of Eq. (3) with *d* = 1.75 can be barely resembled by two 2 × 2 matrices. The enhancement in lifetime (defined as *max*(*Im*(*E*_1_)/*Im*(*E_i_*))) by the 3 × 3 matrix is also around 2. This value is similar to the effect of individual strong coupling and indicates the weak interaction between two strong couplings at larger separation distance.

Once the separation parameter d is small enough, e.g. *d* = 0.225, the behaviors of the three-state model becomes quite different. In additional to the anti-crossings in real parts (see Fig. 1(c)), the imaginary part of state-2 has been dramatically increased to ~ 0 around Δ = *d*/2. The corresponding lifetime enhancement factor is more than 10^4^. The inset in Fig. 1(d) summarizes the influence of *d* on the enhancement factor, where the enhancement in lifetime can be improved by several orders of magnitude (with *Im*(*E*) → 0). Therefore, we know that the interaction between strong couplings can dramatically amplify their influences on lifetime although the individual effect of strong coupling is weak.

### CDT in chaotic microcavity

We then test our analysis with optical microcavities, whose resonances and frequencies play the roles of states and their energies in quantum systems^17^,^20^,^21^,^22^,^23^. Chaotic cavities are selected here due to their mixed phase space structures^17^,^18^,^20^,^21^,^22^,^23^,^24^. When the cavity shapes are not largely deformed, there are usually several regular states such as stable islands that are surrounded by the chaotic sea. The leakages of long-lived resonances within these regular states consist of direct tunneling and dynamical tunneling, which is also known as chaotic assisted tunneling (CAT). By comparing the computed Q factors and the estimations of direct tunneling and CAT, the latter has been pointed out to be the main channel to dominate the decay of chaotic microcavities^25^,^26^. In 2010, Shinohara et al have experimentally verified such dominance^24^ by measuring the far field laser emissions along the unstable manifolds^27^,^28^. All these researches give very clear relationship between Q factors and tunneling rate^19^. The changes in tunneling rate closely relate to the leakages and thus can be monitored by Q factors. Therefore, chaotic microcavities can be nice platforms to test and apply Eq. (3) in tunneling problems.

**Figure 2:**
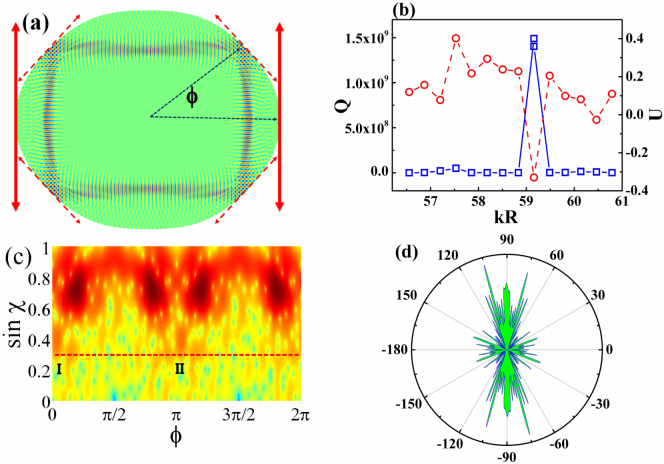
(a) The schematic picture of the oval shaped microcavity. The solid and dashed arrows correspond to the refractive escape along unstable manifolds and the evanescent tunneling along the tangential lines^24^, respectively. (b) The Q factor (open squares) and directionality U (open circles) as a function of kR. (c) and (d) are the Husimi map and far field pattern of the resonance at *kR* ~ 58.13.

The cavity shape studied in this letter is schematically shown in Fig. 2(a). It is a well-known oval shape^24^, which is defined in polar coordinates as *ρ*(*ϕ*) = *R*(1 + *ε*_1_*cos*2*ϕ* + *ε*_2_*cos*4*ϕ* + *ε*_3_*cos*6*ϕ*), where R and *ε_i_* are the size and deformation parameters. Below, without specific explanation, all the deformation parameters are *ε*_1_ = 0.1, *ε*_2_ = 0.01, *ε*_3_ = 0.011. The ray dynamics within such cavity has been thoroughly studied before and only a period-4 stable islands remains above the critical line^18^,^24^. Due to the wave-ray correspondence, the long-lived resonances along stable islands are the rectangle modes as schematically shown in Fig. 2(a)^24^,^25^.

We then numerically calculated the transverse electric polarized (TE, E is in plane) resonances in the cavity by using the finite element method^29^. Here we set the refractive index *n* = 3.4. The calculated results are shown as open squares in Fig. 2(b). Within a wide range of kR, we can find a set of long-lived resonances with equal mode spacing. Most of the quality (Q, *Q* ~ *ωτ*, where *ω* is angular frequency) factors are around the 10^5^–10^7^. Only the resonance at *kR* ~ 59.16 have extremely high Q factors more than 10^9^, which is orders of magnitude higher than the others. While the dynamical tunneling can vary with the nkR, such a difference is still surprisingly high.

From the field patterns, we know that the mode at *kR* ~ 59.16 and the other high Q resonances in Fig. 2(b) are confined along the same rectangle orbit (see an example in Fig. 2(a)), which corresponds to the period-4 stable island above the critical line^18^,^24^. Then the emissions of such long-lived resonances are supposed to be similar too. According to previous researches^24^, two types of decay channels relate to the rectangle modes along stable islands. One is the direct tunneling to critical line and the other one is the CAT^24^. Due to the small tunneling distance between stable islands and the surrounding chaotic sea, the main leakage is dominated by the CAT. Then the outputs of such high Q modes are supposed to follow the refractive escapes along the same set of unstable manifolds^24^,^27^ and form bi-directional emissions along *ϕ_FF_* = ±90°^18^,^24^.

**Figure 3:**
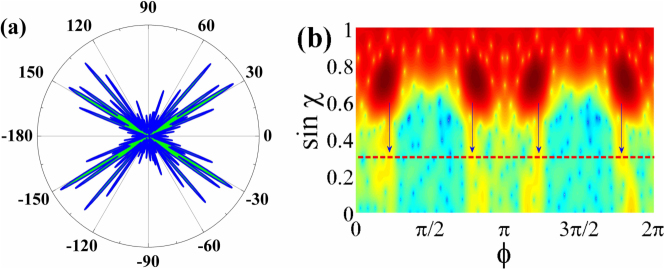
The far field pattern (a) and Husimi map (b) of the resonance at *kR* ~ 59.16. Different from the nearby resonance, here the far field pattern contains four directional beams formed by the direct tunneling.

To qualitatively characterize the directional outputs (relates to the CAT), we define a measure 
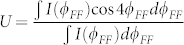
. The results are plotted as open circles in Fig. 2(b) too. We can see that most of the long-lived resonances have positive U, which relates the emissions along the *ϕ_FF_* = ±90° (see an example in Fig. 2(d)) and is consistent with the prediction of universal emissions^24^. Again, the drastic change is found at *kR* ~ 59.16. The U factor of the resonance with extremely high Q dramatically reduces to negative value ~ −0.33. The corresponding far field pattern in Fig. 3(a) shows that it mainly consists of four emission peaks *ϕ_FF_* = ±45°, 135°. The conventional bi-directional emissions along *ϕ_FF_* = ±90° have been nearly completely suppressed.

## Discussion

The coincident changes in Q factors and far field patterns at *kR* ~ 59.16 are similar to the “locking” effect near level crossings, which is the complete suppression of tunneling in CDT^18^. For the long-lived resonances within stable islands, the direct tunneling will dominate the decay channel if the CAT is suppressed significantly. Due to the larger barriers of direct tunneling, the light confinement (and Q factors) will be dramatically improved as Fig. 2(b). Most importantly, the far field emissions generated by the direct tunneling should follow the evanescent escape along the tangential lines. As the dashed lines depicted in Fig. 2(a), such evanescent escapes give four directional outputs along *ϕ_FF_* = ±45°, 135°, agreeing Fig. 3(a) very well.

The above picture can be further confirmed by the Husimi map, which is a projection of wave functions on the cavity boundary into the phase space by using the Husimi Functions^30^,^31^. The Husimi maps give more detail information of resonances in terms of incident angle and positions, which correspond to the vertical and horizontal axes, respectively. For most of the resonances, we can see that their main field distributions are localized in the period-4 stable islands and their emissions along the unstable manifolds reach the critical line at *ϕ* = 0, *π* (see the marker I, II in Fig. 2(c) as an example). All these results are consistent with the rectangle orbit (Fig. 2(a)) and emissions along *ϕ_FF_* = ±90° very well (see Fig. 2(d)). However, the Husimi projection of the mode at *kR* ~ 59.16 gives quite different results (Fig. 3(b)). While the resonance is still confined within the period-4 islands, its leakages below the critical line are significantly different from Fig. 2(c). Four peaks in Husimi map (marked by the vertical arrows) can be observed right below the stable islands and the emissions at *ϕ* = 0, and *ϕ* = *π* (along the unstable manifolds) in Fig. 2(c) are suppressed to almost zero. Such changes in Husimi maps below the critical lines match the far field patterns well and clearly demonstrate the transition from CAT to direct tunneling. As the CAT is usually orders of magnitude larger than direct tunneling^25^,^26^, we thus can conclude that the dynamical tunneling (or CAT) is nearly completely suppressed, similar to the locking in CDT.

**Figure 4:**
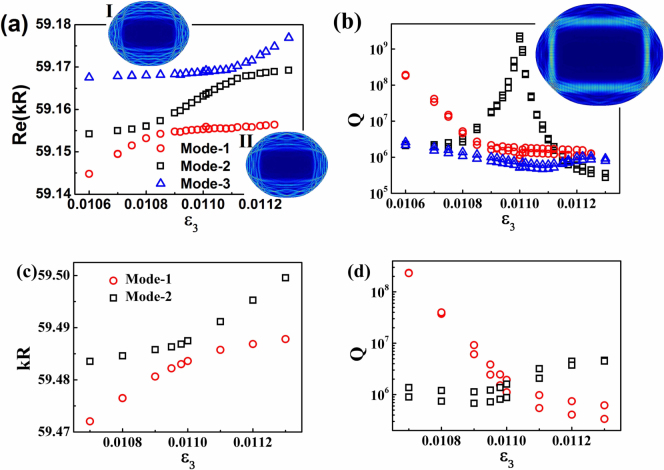
The calculated kR (a) and Q (b) factors of the resonance at kR 59.16 as a function of shape deformation at kR 59.16 as a function of shape deformation *ε*_3_. Two anti-crossings can be found at *ε*_3_ ~ 0.01085 and 0.01115, respectively. The maximum Q factor happens at *ε*_3_ ~ 0.011, which is close to the center of two anti-crossings. The insets I and II are the field distributions of chaotic modes nearby the rectangle resonance. (c) and (d) show the dependence of kR and Q factors of mode-1 and mode-2 on *ε*_3_. All the resonances are nearly degenerated doublets which have odd and even symmetries with respect to the axis *ϕ* = 0.

Now the intriguing question is how the locking is formed. Besides the light confinement along the rectangle orbit, the field pattern of mode at *kR* ~ 59.16 also shows additional distributions along the cavity boundary (see inset in Fig. 4(b)). Then an intuitive picture is that the CAT is fully suppressed by the CDT around level crossing as Ref. 10, 18. We then study the nearby low Q resonances around the high Q mode at *kR* ~ 59.16 to verify this hypothesis. Two sets of low Q resonances have been found around the extremely high Q resonance. Their field distributions (see inset I and II in Fig. 4(a)) indicate that they are the chaotic modes formed by the wave localizations. Such chaotic modes have different mode spacing from the rectangle resonance and approach the rectangle mode individually, indicating the possibility of mode coupling too.

To get the inside view of mode interactions, we have studied their resonant frequencies and Q factors as a function of shape deformation. The results are summarized in Fig. 4 (a) and (b). With the decreasing of deformation parameter *ε*_3_ from 0.011 to 0.0106 (the other parameters are the same as above), we can see that mode-1 and mode-2 approach each other and then repel at *ε*_3_ ~ 0.01085. And their imaginary parts show an obvious crossing. Meanwhile, we have observed the exchange in field distribution before and after anti-crossing point, confirming the occurrence of strong coupling instead of weak coupling^10^. Similarly, strong coupling is also observed between mode-2 and mode-3 at *ε*_3_ ~ 0.01115, indicating the possibility of new mechanism for CDT and “locking”.

Prior to the three-state model, we first test the influence of individual anti-crossing on CAT. We switch the normalized frequency from *kR* = 59.19 to *kR* = 59.49, where mode-1 and mode-2 couple with each other and mode-3 is separated far away. Figure 4 (c) and (d) show the dependencies of kR and Q factors on the shape deformation *ε*_3_. Distinguishing characteristics of strong coupling can be observed in Fig. 4(c) and (d). Interestingly, while the changes of Q factors on the left side of anti-crossing is similar to the interaction between mode-1 and mode-2 in Fig. 4(b), their behaviors on the right side of anti-crossing point (with Δ*ε*_3_ ~ 0.0003) are quite different. The Q factors of long-lived resonances are only slightly enhanced in Fig. 4(d). The further calculations in far field patterns also confirm that no locking happens around the anti-crossing. all these infomration also hold true for the strong coupling between mode-1 and mode-3. Thus we know that the locking around *ε*_3_ = 0.011 in Fig. 4(b) should not be attributed to the two-state strong coupling.

From the combination of highest Q mode and its Husimi pattern (see Fig. 3(b)), we know that the locking happens at the center between two anti-crossings, bearing strong similarity with the behaviors in Fig. 1(c) and (d). Then the inhibition of chaotic assisted tunneling becomes understandable after taking the interaction between two strong couplings. While the tunneling is slightly influenced by two individual strong coupling, the tunneled light along different chaotic orbits can have interaction at the center of two anti-crossings due to the spectral overlap. Meanwhile, the universal emission along the same sets of unstable manifolds^24^,^27^ ensures the spatial overlapping. Thus the relative phases turn to be essential. Once the leakages along unstable manifolds interfere destructively, the emission along this decay channel will be suppressed. Then the tunneling from stable islands to chaotic sea and its reversed process will reach a balance and the distribution within the chaotic sea is almost frozen like the locking in CDT. Interestingly, the light cannot be trapped infinitely inside the open system. Once one decay channel is blocked, the main leakage will shift from to another one. In oval shaped cavity, such change is consistent with the transition from bi-directional emissions (Fig. 2(d)) to four directional emissions (Fig. 3(a))in far field pattern, giving a new criterion to observe complete suppression of CAT experimentally^24^.

It is worth to note the difference between this mechanism and dynamical localization in optical microcavities^32^. While both of them rely on the destructive interference along different paths^33^, three-state anti-crossings model can reach the locking point, where the dynamical tunneling is fully suppressed. Thus the Q factor can be orders of magnitude higher and the far field emissions are switched. Moreover, compared with unpredictable multi-paths in dynamical tunneling, the properties of three resonant modes are all known and much easier to predict and design.

In summary, we have studied the impacts of anti-crossings on the CDT. In additional to the conventional CDT around the level crossings in a two-state model, we show that the CDT can also be formed by the anti-crossings in a three-state model. While the individual influences of strong couplings on the tunneling are negligible due to their moderate coupling constant, the interactions between two strong couplings can significantly suppress the tunneling. In an oval-shaped microcavity, we show that the dynamical tunneling is full inhibited by the destructive interference between two strong couplings. Our results are not limited in the research of semiconductor microcavities, they can also find their applications in the coherent control of quantum tunneling in other open systems e.g. coherent population trapping^34^, non-absorption resonance^35^, cavity QED^11^, and dark states^36^.

## Methods

As the thicknesses of microdisks are much smaller than their in-plane dimensions, microdisks are usually treated as two-dimensional objects by applying effective refractive indices *n*. Then the wave equations for transverse electric (TE, E is in plane) polarized modes *H_z_*(*x*, *y*, *t*) = *ψ*(*x*, *y*)*e*^−*iωt*^ can be replaced by the scalar wave equation 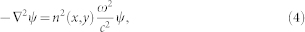
with angular frequency *ω* and speed of light in vacuum *c*. We numerically computed the TE polarized resonances by solving above equation with the RF module in COMSOL Myultiphysics 3.5a. The cavity shape is defined with AutoCAD and imported to the software. And the Q factor is determined by *Q* = *Re*(*ω*)/2|*Im*(*ω*)|.

## Author Contributions

Q.S. and S.X. designed the research. Q.S., S.L., Z.G. performed the numerical calculation and analysis. Q.S. and S.X. wrote the manuscript and all authors reviewed the contents.”.
